# Exosomes in Prostate Cancer Diagnosis, Prognosis and Therapy

**DOI:** 10.3390/ijms21062118

**Published:** 2020-03-19

**Authors:** Tomasz Lorenc, Katarzyna Klimczyk, Izabela Michalczewska, Monika Słomka, Grażyna Kubiak-Tomaszewska, Wioletta Olejarz

**Affiliations:** 1 1st Department of Clinical Radiology, Medical University of Warsaw, 02-004 Warsaw, Poland; 2 Department of Biochemistry and Pharmacogenomics, Faculty of Pharmacy, Medical University of Warsaw, 02-097 Warsaw, Poland; kasiaklimczyk6@gmail.com (K.K.); izabela1356@gmail.com (I.M.); slomka.monika.94@gmail.com (M.S.); grazyna.kubiak-tomaszewska@wum.edu.pl (G.K.-T.); wolejarz@wum.edu.pl (W.O.); 3 Centre for Preclinical Research, Medical University of Warsaw, 02-097 Warsaw, Poland

**Keywords:** extracellular vesicle, precision oncology, cancer biomarker, prostate cancer

## Abstract

Prostate cancer (PCa) is the second most common cause of cancer-related mortality among men in the developed world. Conventional anti-PCa therapies are not effective for patients with advanced and/or metastatic disease. In most cases, cancer therapies fail due to an incomplete depletion of tumor cells, resulting in tumor relapse. Exosomes are involved in tumor progression, promoting the angiogenesis and migration of tumor cells during metastasis. These structures contribute to the dissemination of pathogenic agents through interaction with recipient cells. Exosomes may deliver molecules that are able to induce the transdifferentiation process, known as “epithelial to mesenchymal transition”. The composition of exosomes and the associated possibilities of interacting with cells make exosomes multifaceted regulators of cancer development. Extracellular vesicles have biophysical properties, such as stability, biocompatibility, permeability, low toxicity and low immunogenicity, which are key for the successful development of an innovative drug delivery system. They have an enhanced circulation stability and bio-barrier permeation ability, and they can therefore be used as effective chemotherapeutic carriers to improve the regulation of target tissues and organs. Exosomes have the capacity to deliver different types of cargo and to target specific cells. Chemotherapeutics, natural products and RNA have been encapsulated for the treatment of prostate cancers.

## 1. Introduction

Prostate cancer (PCa) is the most common solid malignancy, with a high mortality in men [1]. In many cases, successful treatment of prostate cancer is difficult due to the late detection and rate of metastasis [2]. Importantly, the tumors of many patients with prostate cancer become refractory to androgen therapy and progress to metastatic castration-resistant disease [3]. An effective treatment course of prostate cancer patients requires predictive biomarkers in metastatic castration-resistant prostate cancer that support individual therapy [4]. Liquid biopsies, circulating tumor cells, exosomes and circulating nucleic acids have been developed as minimally invasive assays to monitor PCa patients [5]. Exosomes are extracellular vesicles (EVs), which may serve as novel tools for various therapeutic approaches, including drug delivery [6], anti-tumor therapy, pathogen vaccination, immune-modulatory and regenerative therapies. They are secreted by cells and detected in various biological fluids, and they can serve as biomarkers for cancer diagnosis, prognosis and therapy [7]. The presence of EVs in urine was discovered in 2004 [8]. It is believed that urinary EVs originate from epithelial cells of the urogenital system, which includes the organs involved in reproduction and urine excretion. Urine has additional advantages relating to cancers of the urogenital system, since the composition of urine directly reflects changes in the function of associated organs. Blood-specific prostate antigen (PSA) remains the most widely used biomarker in the detection of early prostate cancer, but new biomarkers, like exosomal miRNAs, have been proposed to increase specificity and distinguish aggressive from non-aggressive PCas [9]. It has been shown that urinary markers can aid in the decision-making process regarding whether to carry out a prostate biopsy and in the design of a therapeutic strategy [10]. Urinary exosomes and their cargo, especially miR-21 and miR-375, have become an emerging source of biomarkers in the detection and prognosis of PCa [11]. Moreover, the expression of serum exosomal miRNAs induced by radiotherapy may have potential value as prognostic and predictive biomarkers PCa [12]. Exosomes are also promising carriers of drugs and other therapeutic molecules targeting prostate cancer, but there are still several challenges relating to their use as drug carriers [13]. 

## 2. Structure and Function of Exosomes

**Figure 1:**
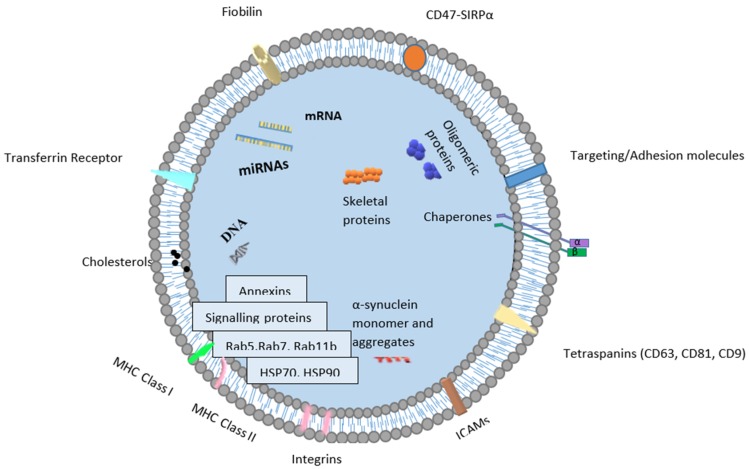
Schematic diagram of an exosome.

Exosomes are small (from 30 to 120 nm in diameter) extracellular vesicles (EVs) (Figure 1). Their lipid bilayer membrane, with a width of 5 nm, protects them from the negative action of RNases and proteases. Exosomes have a longer retention in circulation in comparison to polymersomes or liposomes [14]. This characteristic originates from transmembrane protein (CD47-SIRPα), which prevents exosomes from being phagocytosed [15]. Moreover, the double membrane structures contain various cargo, such as miRNAs, proteins (e.g., tetraspanin CD63, CD81, CD82, CD53 and CD37, as well as cystolic proteins), lipids (e.g., sphingomyelin, cholesterol and generally saturated fats) and viral particles [16], and their presence depends on the origin cells and organism’s health conditions. The proteins in exosomes include endosomal, plasma and nuclear proteins [17]. Therefore, in comparison to cellular proteins in prostate cancer, those in exosomes have a higher level of glycosylation [18]. Moreover, proteins enriched in exosomes include those relevant for individual exosomal biogenesis pathways and for exosome secretion. The Minimal Information for Studies of Extracellular Vesicles 2018 highlights three categories of markers that must be found in isolated extracellular exosomes. They include at least one transmembrane/lipidbound protein or cytosolic protein and one negative protein marker [19]. The cargo present in extracellular vesicles can be transferred and alter signaling pathways in recipient cells [13]. For example, exosomes from metastatic prostate cancer cells showed high contents of miR-21 and miR-141, which are responsible for the regulation of osteoclastogenesis and osteoblastogenesis. Cancer-derived exosomes can promote epithelial–mesenchymal transition (EMT) via miRNAs. They play an important role in the conversion from benign to malignant cancers [13] and in the regulation of the response to docetaxel, such as miR-34 in prostate cancer cells and cell-derived exosomes targeting Bcl-2. This shows the great influence of extracellular vesicles on the drug resistance of prostate cancer cells. 

**Figure 2:**
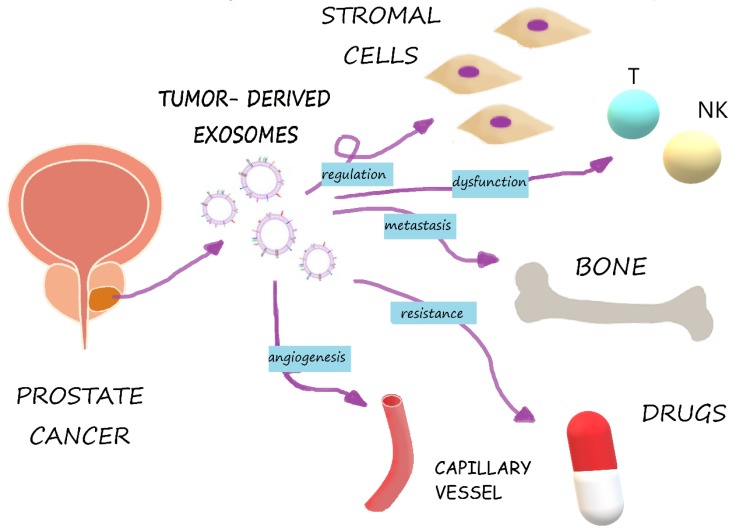
Role of prostate cell-derived exosomes in cancer progression. Exosomes regulate stromal cells, impair immune cells and alter the microenvironment, which could lead to tumor growth and metastasis. Exosomes are also responsible for drug resistance.

Exosomes are released by the exocytosis of multivesicular bodies (MVBs), developed from early and then late endosomes [20]. Those naturally occurring membrane particles mediate intercellular communication by delivering molecular information between cancer and stromal cells, especially cancer-associated fibroblast (CAFs) [14]. Cancer cell-derived EVs cause very diverse effects and may depend on their target cells, which appear to take them up in several ways, such as receptor/lipid raft endocytosis, phagocytosis, micropinocytosis or fusion with the plasma membrane [17]. Some exosomes can reduce the anti-cancer immune response, interact with specific membrane receptors [9] and promote a suitable microenvironment, while others may cause drug resistance and the failure of antibody therapy involving RNA species and protein delivery [21]. Recent studies point out that exosomes released from the tumor microenvironment can regulate (also by tethering TGFβ) a proliferation, a reduction of apoptosis, a promotion of angiogenesis and, finally, an evasion of immune surveillance (Figure 2). Moreover, exosomes can provide candidate biomarkers for prostate cancer, contribute to tumor progression and, after a loss of environment homeostasis, promote tumor metastasis [18]. 

## 3. Tumor-Derived Exosomes in Cancer Progression

### 3.1. Tumorigenesis

Tumorigenesis or carcinogenesis is the process of an uncontrolled multiplication of cells or deregulated apoptotic cell death, which leads to the formation of cancer. Exosomes derived from prostate cancer cells, which have become the common object of scientists’ interest, are implicated in creating a premetastatic niche [13]. It is a microenvironment that is especially favorable to cancer cells composed of different cell types, like fibroblasts, lymphocytes, epithelial cells, matrix molecules, factors, such as growth factors, and cytokines [22]. Exosomes influence the immune system in a premetastatic niche through various mechanisms. Prostate cells are involved in producing tumor-derived secreted factors (TDSFs), including VEGF, TNF-α and interleukins in response to the local inflammatory niche. First, TDSFs stimulate the recruitment of myeloid cells and immune cells to the pre-metastatic niche. Furthermore, the expression of inflammatory factors is upregulated by stromal cells under the influence of TDSFs. Moreover, tumor-derived exosome content enhances the pre-metastatic niche [23]. Tumor exosomes possess the capacity to support the migration of immune cells, like neutrophils, macrophages and regulatory T to secondary sites, thereby reducing immune response against tumors and inhibiting antigen-presenting cells, such as dendritic cells. These nanoparticles could impair the function of T-cells and NK-cells via the blocking, proliferation, activation and provision of apoptosis [23,24]. 

### 3.2. Tumor Progression

Recent research suggests that exosomes isolated from the prostate cancer microenvironment are an important factor in the progression of this type of cancer. The increase in the mass of prostate tumors may be the result of tumor stem cell proliferation, which possess a self-renewing ability [13,25]. Prostate cancer exosomes, as the carriers of many lipids, proteins and RNAs, can affect the proliferation, angiogenesis and survival of cancer cells, as well as their ability to avoid immune surveillance [26]. Exosomes, which form in the tumor microenvironment, transport, among other things, miRNAs (miRs, short noncoding RNAs that are responsible for the regulation of gene expression). miR-20a, miR-21 and miR-125b cause the inhibition of apoptosis and survival, promoting the effects of cancer cells. miRNA-221 and miR-222 are responsible for cancer growth. miR-92a and miR-17-92 are involved in the promotion of angiogenesis, as a result of an increased proliferation and migration of endothelial cells. It has been shown that miR-210 is responsible for the induction of metastasis by EMT promotion. miR-21, miR-100 and miR-139 induce fibroblast migration by increasing the expression of MMP-2, MMP-9, MMP-13 and RANKL. miR-21, miR375 and miR-141 overcome low androgen conditions during distant metastasis. miR-1290 and miR-375 are connected with a poor patient prognosis. miR-409 downregulates tumor suppressors, like RSU1 and STAG2, and promotes cancer cell tumorigenesis. The ANXA6/LRP1/TSP1 complex causes tumor growth induction. miR-126 and miR-146a are involved in tumor suppression [27,28,29,30]. Moreover, exosomes, with a structure rich in lipids, especially cholesterol and sphingomyelin, can modulate the lipid composition of the target cells, disturbing their homeostasis. A study on prostate cancer has shown that the accumulation of cholesterol esters in prostate cancer cells is associated with tumor progression and metastasis. This was confirmed by studies, using synthetic exosomes carried out by the Lombardo Group, which showed that exosomal lipids can increase the tumor aggressiveness, metastatic progression and drug resistance of pancreatic cancer cells [31]. 

It is well known that exosomes secreted by prostate cancer cells under hypoxic conditions increase the invasiveness, mobility and EMT in native PC cells and promote the transformation of fibroblasts into myofibroblasts. Moreover, under these conditions, lipid accumulation (triglycerides, bis-monoacylglycerolphosphate, ceramides, cholesterol, etc.) increase in PCs, which leads to increased exosome secretion by hypoxic tumor cells [32].

Exosomes also participate in the regulation of the progression of prostatic tumors, as the carriers of numerous proteins. These vesicular structures, as carriers of TGF-β, can induce the above-mentioned transformation of fibroblasts into myofibroblasts (CAFs) by the activation of TGF-β/Smad3 signaling or independent SMAD signaling pathways and promote neoangiogenesis. Webber found that exosomal TGFβ1 induces a highly aggressive myofibroblast phenotype, with a high proangiogenic activity [13,33]. ITGA3, ITGB1, ITGB4 and ITGB3 are exosomal proteins involved in the progression of prostate cancers. These integrins are responsible for the promotion of the migration and invasion of epithelial cells by the activation of Src phosphorylation in recipient cells. The matrix metalloproteinases MMP-9 and MMP-14 promote cancer cell protection from apoptosis and intensify their mobility through the stimulation of ERK1/2 phosphorylation. Subsequently, they prepare the metastatic site [26,27]. The next group, ligands for the NKG2D and Fas receptors, causes tumor immune evasion by receptor downregulation and silences the cytotoxic activity of NK cells and CD8+ cells. Caveolin-1 increases the survival of cancer cells and their independence from androgens, and they also promote distant metastasis by the positive regulation of fatty acid synthase activity. Hypoxia-inducible factor 1α (HIF-1α) promotes the initiation and progression of metastasis by promoting the loss of E-cadherin. The tetraspanin–integrin complex increases the adhesion of exosomes to the right cells. The epidermal growth factor receptor (EGFR) induces tumor angiogenesis by the activation of the autocrine VEGF/VEGFR-2 pathway in endothelial cells. Tyrosine-protein kinase Met induces metastasis and promotes a phenotype resistant to castration therapy. Other exosomal proteins, c-Src tyrosine kinase, IGF-1R and FAK, induce angiogenesis by the stimulation of the VEGF transcription within the tumor microenvironment [34,35,36,37,38,39].

It has also been shown that tumor-derived exosomes enhance interleukin-6 production in myeloid-derived suppressor cells through the activation of Toll-like receptor 2 via the membrane-linked heat shock protein, which promotes the autocrine phosphorylation of Stat3 and enhances the effect of the immunosuppression of the immune system and the promotion of prostate cancer [40].

Exosomes secreted by prostate cancer cells are also involved in tumor metastasis in bone, which is common in patients with this type of cancer. Recent studies have shown that prostate cancer cell-derived exosomes mediate cell–cell communication in osteoblastic metastasis. On the other hand, osteoblast-derived exosomes may regulate prostate cancer cell proliferation at the original site of tumor development [41].

Exosomes secreted by tumor cells affect the stromal cells by stimulating the formation of pro-proliferative and proangiogenic phenotypes in these cells. It has been shown that exosomes secreted by cancer-associated fibroblasts increase the ability of prostate cancer cells to proliferate and survive in low-oxygen and low-nutrient environments by inhibiting mitochondrial oxidative phosphorylation and increasing anaerobic glycolysis [33,42].

### 3.3. Angiogenesis

Exosomes obtained from prostate cancer stem cells support tumorigenesis by promoting angiogenesis, which, as a process of developing a new vasculature, is crucial for tumor growth and migrations and is the main cause of metastasis and malignancy. A recent report has shown that some conditions, like hypoxia or acidosis, enhance secreting exosomes in bodily fluids, and these exosomes cause angiogenesis more frequently [30]. Studies have also reported that the expression of E-cadherin and carbonic anhydrase 9 in exosomes could contribute to the angiogenesis process [43]. In the case of prostate cancer, the process of vascularization is supported by the transfer of sphingomyelin and CD147 via exosomes into endothelial cells [44]. Newly formed blood vessels are created to facilitate tumor growth by transporting TDSFs and circulating tumor cells (CTCs) into secondary tissues, thereby beginning vascular leakage [23]. Prostate cancer cell-derived exosomes, via the activation of TGF-β\SMAD3 signaling, lead to the transformation of fibroblasts into myofibroblasts, referred to as CAFs. The exosomes derived from these special kinds of cells are able to cause an explosive growth of prostate cancer cells by transferring the miRNAs (miR-21 and miR-409) into neighboring epithelia. miR-21 suppresses the expression of APAF1 (apoptotic peptidase activating factor 1) and PDCD4 (programmed cell death 4) to inhibit apoptosis and make cancer cells resistant to chemotherapeutics [13]. Increased angiogenesis and vascular permeability promote metastasis. 

### 3.4. Metastasis

Tumor metastasis is a complicated process, including vascular leakiness and an alteration of the microenvironment, in which exosomes are also involved. Initially, exosomes begin an epithelial–mesenchymal transition (EMT) via miRNAs by losing their junction and adhesion ability. Thus, epithelial tumor cells obtain mesenchymal cell properties and are responsive to malignancy [45]. Exosomes support the formation of a pre-metastatic niche, and cells could then be found at the vascularized organs, but metastasis cannot be developed randomly, but rather only in preferential sites under the direction of exosomes. In the process of this expansion, the special kind of CTCs (metastases-initiating cells (MICs)) are involved [46]. MICs can act on other cells by secreting exosomes that reprogram adjacent stromal cells to create a more favorable tumor microenvironment in order to support cancer growth and progression. As mentioned above, cancer-derived exosomes determine organotropism. Prostate cancer possesses an affinity with bone. Exosomes obtained from prostate cancer cells by osteoclast fusion and differentiation support transmission to this destination [47]. Furthermore, exosomes derived therefrom, through the activation of RANKL, FOXM1 and c-Myc, support EMT [46]. As described previously, cancer cell-derived exosomes transfer various substances, including integrins, which are responsible for organotropism. Integrin σ3 and β1 lead to the migration and dissemination of epithelial cells. In addition, integrin avb6, expressed on the surface, is responsible for the metastatic phenotype [48]. The tumor microenvironment contributes to the regulation of prostate cancer progression through proliferation, angiogenesis and metastasis, and it also regulates immunity.

### 3.5. Tumor Immune Escape

The immune system is able to recognize transformed cells and eliminate them. Thus, tumors use diverse mechanisms to escape from immune-mediated surveillance. Exosomes derived from prostate cancer cells impair the cytotoxic function of lymphocytes and induce the apoptosis of CD8+ T cells [23]. In the first step, exosomes activate the T cell receptor, and the expression of Fas on the T cell is then upregulated. Subsequently, FasL induces apoptosis directly via receptor CD 95/APO1 or indirectly using dendritic cells [49]. Fas-mediated apoptosis, as an immune-evasive mechanism, may lead to tumorigenesis, but it may also be responsible for drug resistance [49]. Some other mechanisms and molecules may also contribute to T cell apoptosis. Among the substances carried by exosomes is the programmed death ligand 1 (PDL-1). Exosomal PDL-1 plays the same role as tumor PDL-1. PDL-1 binds to its receptor, PD-1, expressed on the surface of activated T and B cells or macrophages, and enables T cell apoptosis [50]. On the other hand, cancer-derived exosomes can not only cause the dysfunction of T and NK cells by blocking the activation and proliferation or induction of apoptosis, but also inhibit antigen-presenting cells and antitumor immune response [23]. While exosomes are characterized by heterogeneity and a risk of developing metastasis, those nanoparticles could be used as diagnostic and prognostic biomarkers for various cancers and as promising drug carriers. 

## 4. The Potential of Exosomes in Prostate Cancer Diagnosis and Monitoring

EVs may be key biomarkers in the early diagnosis of prostate cancer and in a personalized approach to treatment and to future patients’ prognosis [26]. EVs in the blood and urine of prostate cancer patients contain unique prostate-cancer-specific contents, which are biomarkers of prostate cancer and cancer metastasis [51,52]. Exosomes present many different proteins on their surface. These proteins can act as epitopes, which are recognized using different mono- or polyclonal antibodies. A dedicated assay has been established to determine if the exosomes in blood plasma can serve as markers for prostate cancer [51]. Additionally, several EV-derived proteins have been investigated as potential cancer biomarkers in clinical settings [53]. Several urinary exosome proteins showed a high sensitivity and specificity for prostate cancer as individual biomarkers, and combining them in a multi-panel test has the potential for a full differentiation of prostate cancer from non-disease controls [52]. Liu et al. showed that exosomes from prostate cancer are highly enriched with PSA, representing characteristics of the original PCa cells [54]. Current evidence indicates that the strongest candidates for intercellular communications in PCa are exosomal RNAs [55]. The EV-derived RNAs have also been assessed in terms of their potential as cancer biomarkers in clinical samples. Yang et al. confirmed, through meta-analysis, that plasma exosomal miRNAs have a high diagnostic value for prostate cancer patients [56]. Hessvik et al. identified 36 exosomal miRNAs as biomarker candidates for PCa in clinical studies [57]. In prostate cancer, plasma vesicles, isolated using the precipitation-based ExoQuick method, identified miR-1290 and miR-375 as potential prognostic biomarkers in castration-resistant prostate cancer (CRPC), since their level correlates with a poorer overall survival (*p* < 0.004) [58,59]. Joncas et al. demonstrated that the exosomal androgen receptor splice variant (AR-V7) is correlated with lower sex steroid levels and with a poor prognosis in CRPC patients [60]. For prostate cancer, the expression of the AR-V7 RNA in CTCs was identified as a predictive marker for response to enzalutamide (anti-androgen) and abiraterone (anti-androgen and CYP17 inhibitor) [61]. Whether the AR-V7 transcript can also be measured in plasma EVs and serve as a biomarker was investigated by Del Re et al. [62]. Using the exoRNeasy purification method, EV-RNA was extracted, and the AR-V7 transcript was detected, preferentially in patients resistant to enzalutamide or abiraterone treatment. This study, as well as other studies, shows the biomarker potential of plasma-derived EV-RNA and the advantages with respect to the cost, ease of workflow and likelihood that all (heterogeneous) tumors are represented by a specific EV population. Communication between a tumor and its environment in PCa via extracellular vesicle (EV) RNAs is a potential mechanism of bone metastasis [63]. It has been shown that exosomal miR-375 significantly promoted osteoblast activity [64]. Krishn et al. proved that prostate cancer sheds the αvβ3 integrin in vivo through exosomes. The exosomal αvβ3 integrin has been shown to promote aggressive phenotypes in many types of cancers and may be clinically useful as a non-invasive biomarker to follow prostate cancer progression [65]. Another paper has reported survivin as a plasma-derived EV biomarker through the isolation of total EVs by the ultracentrifugation-based method for prostate cancer [66]. It has been shown that exosomes released by irradiated prostate cancer cells are enriched in B7-H3 protein (CD276), which has been identified as a diagnostic marker [67]. Furthermore, the serum exosomes of prostate cancer patients undergoing radiotherapy had increased levels of HSP72, which plays a key role in the stimulation of pro-inflammatory immune responses [68]. Additionally, urinary exosomes are a promising non-invasive biomarker, with a potential use in the diagnosis, prognosis and monitoring of prostate cancer [69]. 

## 5. The Potential of Exosomes in Prostate Cancer Therapy

### 5.1. Exosomes as Drug Carriers for Prostate Cancer Therapy

**Figure 3:**
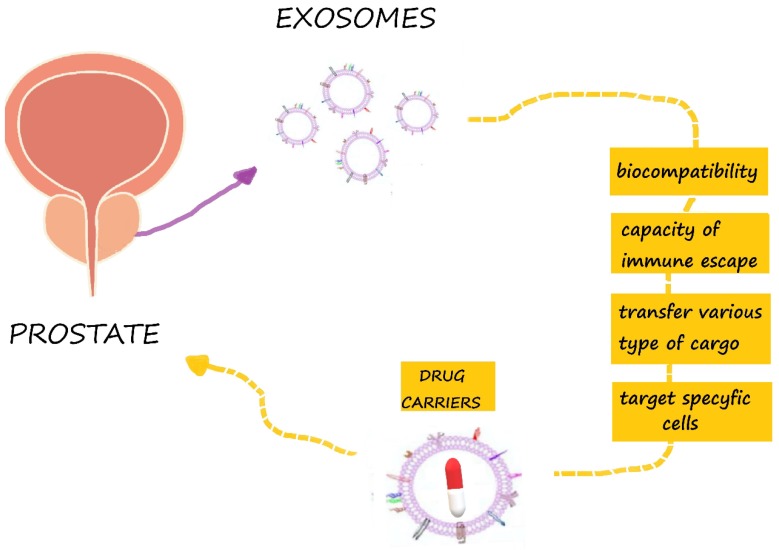
Exosomes are promising drug carriers in prostate cancer therapy.

Extracellular vesicles have biophysical properties, such as stability, biocompatibility, permeability, low toxicity and low immunogenicity, which are key to successful drug delivery systems [70]. They have an enhanced circulation stability and bio-barrier permeation ability, and they can therefore be used as effective chemotherapeutics carriers to improve the regulation of target tissues and organs [71]. Chemotherapeutics, natural products and RNA were combined for the treatment of prostate, breast, pancreatic, and lung cancers, as well as glioblastoma [14]. Exosomes have the capacity to deliver different types of cargo and to target specific cells (Figure 3). They have been tested for the delivery of different therapeutic agents in in vitro and in vivo experiments [72]. EVs can be used as carriers to deliver therapeutic agents to tumor cells, leading to an effective tumor cell killing, while minimizing the side effects of the drugs [73]. It has been shown that mesenchymal stromal cells are able to package and deliver active drugs through their membrane microvesicles (MVs) [74]. Additionally, Tian et al. demonstrated that exosomes modified by targeting ligands can be used therapeutically for the delivery of doxorubicin to tumors [75]. Additionally, Qi et al. confirmed that drug-loaded exosomes enhanced cancer cell targeting under an external magnetic field and suppressed tumor growth [76]. Saari et al. confirmed that cancer cell-derived EVs can be used as effective carriers of Paclitaxel to autologous prostate cancer cells by increasing its cytotoxicity [21]. The simultaneous application of either radiation technology or nuclear medicine with exosomes are promising tools for the realization of the enhancement of targeting strategies using radiation technology [77]. 

### 5.2. Precision Therapy 

Exosomes have been shown to be crucial for the development of drug resistance in patients with prostate tumor. In 2017, Del Re et al. assessed AR-V7 as a predictor of resistance to hormonal therapy by highly sensitive digital droplet polymerase chain reaction in plasma-derived exosomal RNA. They found that both the median progression-free survival (20 vs. 3 months; *p* < 0.001) and overall survival (8 months vs. not reached; *p* < 0.001) were significantly longer in AR-V7-negative vs. AR-V7-positive patients [62]. Exosome-derived microRNAs also contribute to PCa chemoresistance [78] and can act as surrogate biomarkers of tumor response to taxanes [79]. It has been observed that the transfer of exosomes (in particular, MDR-1/P-gp) from docetaxel-resistant cell lines to the DU145, 22Rv1, and LNCap PCa cell lines induces an acquired resistance to this drug [80]. In the same view, Kawakami et al. reported that β4 (ITGB4) and VCL in exosomes could be useful markers of PCa progression, which is correlated with taxane resistance [81]. Interestingly, high serum exosomal P-glycoprotein levels are associated with resistance to docetaxel, but not to cabazitaxel, thus representing a potential biomarker for guiding the decision-making process of PCa patients [82]. 

There is currently also a list of challenges that remain in the era of individualized or person-specific targeting of cancer. EVs are being pursued as intercellular vectors for RNA-based therapy (both miRNAs and siRNAs), with a documented efficacy in animal models of disease. For example, the in vivo suppression of prostate cancer has been achieved through the exosome delivery of the tumor suppressor miRNA, miR-143, in mice [83]. 

## 6. Concluding Remarks and Future Directions

Increasingly, studies confirm the potential of exosomes as therapeutic vehicles for cancer treatment. Exosomes can transfer cargos with both an immunoregulatory potential and genetic information. They can decrease tumor cell invasion, migration and proliferation by enhancing immune response, cell death and sensitivity to chemotherapy. Exosomes have a high potential for both as diagnostic and therapeutic agents in immune therapy, vaccination trials and regenerative medicine. The native structure and unique cellular functions of exosomes give them a great potential as natural drug/gene delivery vehicles, but developing efficient and reliable isolation methods is necessary to fully utilize their potential [84]. The use of exosomes in clinical applications must be further investigated in order to establish standards for exosome characterization and manipulation [85]. The most important thing is to choose optimal methods for engineering exosomes and ensuring the safety of engineered exosomes in clinical trials [86]. Furthermore, the translation of EVs into clinical therapies requires their categorization as active drug components or drug delivery vehicles [6]. It is also necessary to clarify the composition and action mechanism of the various substances in exosomes and determine how to obtain highly purified exosomes and the right dosage for their clinical use [15]. Another important therapeutic use of EVs could be cancer vaccination [87]. In brief, exosomes are very promising tools for the future theranostics of prostate cancer, but their use first requires solutions for the many challenging issues remaining.

## Abbreviations

APAF1	apoptotic peptidase-activating factor 1
AR-V7	androgen receptor variant 7
CAFs	cancer-associated fibroblast
CRPC	castrate-resistant prostate cancer
CTCs	circulating tumor cells
EGFR	epidermal growth factor receptor
EMT	epithelial–mesenchymal transition
EV	extracellular vesicles
FASL	ligand Fas
HIF-1α	hypoxia-inducible factor 1α
HSP	heat shock proteins
ITGB4	integrin β4
MICs	metastases-initiating cells
miR	micro RNA
MMP	matrix metalloproteinase
MVBs	multivesicular bodies
PCa	prostate cancer
PDCD4	programmed cell death 4
PDL-1	programmed death ligand 1
PSA	prostate specific antigen
RANKL	receptor activator for nuclear factor κB ligand
RSU1	Ras suppressor protein 1
siRNA	small interfering RNA
STAG2	cohesin subunit SA-2
TDSFs	tumor-derived secreted factors
TGFβ	transforming growth factor β
VCL	vinculin
